# Recurrent Idiopathic Granulomatous Mastitis: A Case Report

**DOI:** 10.31729/jnma.8442

**Published:** 2024-02-29

**Authors:** Janardan Prasad Joshi, Lila Joshi, Hira Joshi, Ashok Prasad Sah, Thaneshor Neupane

**Affiliations:** 1 Department of General Surgery, Mahakali Provincial Hospital, Mahendranagar, Kanchanpur, Nepal; 2 Lele Primary Health Care Centre, Lele, Lalitpur, Nepal; 3 Baitadi District Hospital, Dasnarathchanda, Baitadi, Nepal; 4 Matrishishu Miteri Hospital, Pokhara, Kaski, Nepal

**Keywords:** *antibiotics*, *case reports*, *granuloma*, *mastitis*

## Abstract

Idiopathic granulomatous mastitis is a rare benign breast disease of unknown aetiology mostly presenting as a breast abscess but not responding to usual conservative management with incision and drainage and frequently mimics breast cancer. We present a case 31-year-old female presented with complaints of right breast pain and redness who was initially diagnosed and treated as a case of breast abscess with repeated incision and drainage and antibiotics but did not improve. Later histopathology revealed granuloma with giant cell reaction and the patient was given a trial of steroids which showed no improvement. Wide local excision with a long course of broad-spectrum antibiotics was performed which led into remission. This case report highlights the importance of considering idiopathic granulomatous mastitis as differentials in non-responding breast abscesses. Histopathology for diagnosis and trial of wide local excision with a long course of broad-spectrum antibiotics as treatment may be done for management.

## INTRODUCTION

Idiopathic granulomatous mastitis is a rare benign breast disease mimicking breast carcinoma.^1^ Although the exact prevalence is unknown, a study has reported a prevalence of 2.4 per 100,000 women aged 20-40 years and 0.37% in the US.^2^ It should be considered as a differential diagnosis when usual therapy for breast abscesses does not cure the disease. There is no pathognomic radiologic finding either on ultrasound or MRI.^3^ A high index of suspicion is key to preventing the morbidity of delayed diagnosis and misguided therapy. There is a great dilemma in its treatment.^4^

## CASE REPORT

A 31-year-old woman presented with on-and-off pain and redness in her right breast for more than a year. There was no history of fever, rash, synovitis, myalgias, lymphadenopathy or other systemic symptoms. Medical, surgical, family and social histories were unremarkable.

The patient was initially diagnosed as a case of mastitis of the right breast based on clinical findings and initial ultrasound but the pus culture grew no bacteria. She underwent treatment with incision and drainage with antibiotics but did not respond to it. She further underwent repeated incision and drainage for the same at various centres without significant response. After 3 months of persistent symptoms despite repeated incisions and drainage and courses of antibiotics, an incisional biopsy was performed that revealed granuloma without any fungal components or atypical cells. A tissue sample for mycobacterium tuberculosis was negative. Evaluation for autoimmune or infectious granulomatous disease was negative, including inflammatory markers, differential cell count, and chest imaging. She was then treated with longterm systemic steroids which was unsuccessful.

Her lab reports were as follows: white blood cell (WBC) count 8500 cells per microliter (Neutrophils = 68%; Lymphocytes = 23%); haemoglobin 14.5 g/dL; serum urea 17.3 mg/dL; serum creatinine 0.9 mg/dL; sodium 130 mEq/L; potassium 3.7 mEq/L. A repeat ultrasound scan of her breast showed features of chronic periductal mastitis with a branching abscess.

**Figure 1:**
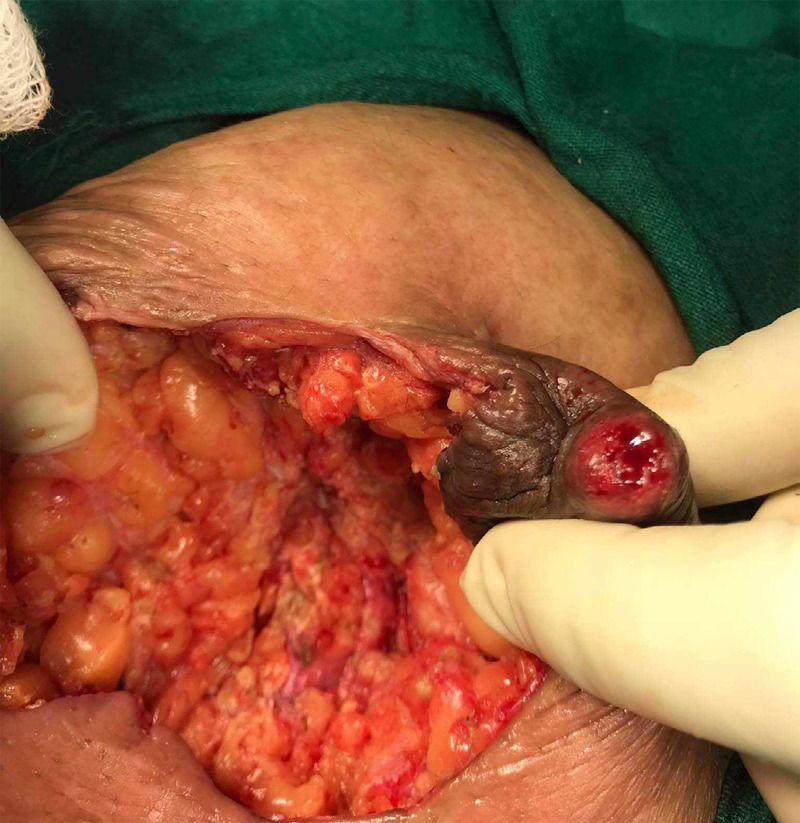
Intraoperative finding showing necrotic material.

The patient was treated with wide local excision with debridement of the necrotic collection found intraoperatively followed by closure (Figure 1).

The excised tissue was sent for histopathological examination which showed acute periductal mastitis with giant cell reaction. There was no evidence of any fungal component on histopathology and acid-fast stain for tubercule bacilli was also negative. She was then presumed to be a case of idiopathic granulomatous mastitis.

**Figure 2:**
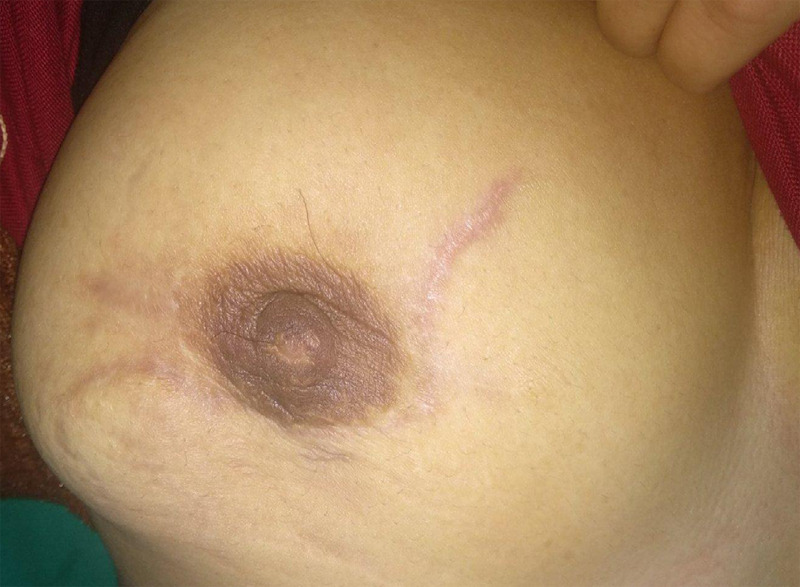
Picture showing well-healed scar at present after 8 months.

She was prescribed a long course of broad-spectrum antibiotics (piperacillin-tazobactam 4.5 gm with metronidazole 500mg both given intravenously and 8 hourly for 2 weeks) following excision. She was then further discharged on cefpodoxime-clavulanic acid 200 mg + 125 mg with ornidazole 500 mg both given orally twice a day for 5 more days. There was mild pain and minimal collection of fluid after 2 months. Her symptoms have completely resolved and as of 8 months now, she is in remission (Figure 2).

## DISCUSSION

Idiopathic granulomatous mastitis is a rare benign chronic inflammatory condition of the breast that often masquerades as other common conditions like breast abscess, tuberculosis, and breast cancer.^5^ The condition is most common in women of childbearing age.^5^ There are several risk factors like oral contraceptives, autoimmunity, infectious causes, and milk spillage from breast lobules.^3,6^ As there is vague clinical presentation which poses a great challenge for diagnosis. It may initially present as breast pain, erythema, induration, lump, ulceration, fistula, and nipple retractions.^5^

In this case also patient is also of childbearing age and presented with symptoms of breast pain and erythema which mimicked breast abscess. It is spontaneous to think of breast abscess when a patient presents with similar complaints like this female but it is of the same vital importance to consider idiopathic granulomatous mastitis as a differential when a patient with clinically apparent breast abscess does not respond to incision and drainage with antibiotics as in this case. Sometimes the presentation of the disease can mimic breast cancer when they present with features of nipple retraction.^5^ It poses a great burden of morbidity due to difficulty in diagnosis and treatment as this female had undergone multiple incision and drainage procedures with antibiotics suspecting it a breast abscess but she did not respond to the treatment.

There is no pathognomic radiologic finding either on ultrasound or MRI which poses a great burden to diagnosis and misdiagnosis as in this case.^3^ In this case, the lady was initially suspected of having breast abscess based on clinical and radiologic evidence showing collection. It is usually a diagnosis of exclusion. The diagnosis is primarily based on histopathologic findings of non-caseating granuloma.^4^ It shows the importance of considering a differential of idiopathic granulomatous mastitis in a non-responding breast abscess. A histopathological examination of the breast tissue was performed that revealed granuloma with a giant cell reaction. It should be kept in mind to rule out tuberculosis and fungal infections in granulomatous mastitis as in this case.

As the aetiology is unclear, the treatment is in dilemma.^4^ Often medical management includes high doses of steroids and antibiotics, as the patient was initially given a trial of systemic steroid with antibiotics but she did not respond to it.^5^ However, literature reviews show that wide surgical excision, corticosteroids, methotrexate, azathioprine, and colchicine can be used for treatment.^3,7^ Treatment depends on the severity of the disease and may include observation, systemic steroids, broad-spectrum antibiotics or surgery. Wide local excision was performed along with a long course of broad-spectrum antibiotics after the failure of systemic steroids. Approximately half of all women have spontaneous resolution without specific therapy.^8^ Those who fail medical management should be considered for wide local excision when there is no remission after long-term conservative management, which happened in this case too.^9^

This case report highlights the importance of considering idiopathic granulomatous mastitis as a differential diagnosis in non-responding breast abscesses. It describes the morbidity related to delayed diagnosis or misdiagnosis and the importance of high clinical suspicion and histopathology for the diagnosis. Clinicians should consider a trial of broad-spectrum antibiotics with wide local excision for the management.
